# On the Role of the Immunoproteasome in Protein Homeostasis

**DOI:** 10.3390/cells10113216

**Published:** 2021-11-18

**Authors:** Michael Basler, Marcus Groettrup

**Affiliations:** 1 Division of Immunology, Department of Biology, University of Konstanz, D-78457 Konstanz, Germany; marcus.groettrup@uni-konstanz.de; 2 Biotechnology Institute Thurgau (BITg), University of Konstanz, CH-8280 Kreuzlingen, Switzerland

**Keywords:** proteasome, immunoproteasome, ubiquitin, ubiquitin–proteasome system (UPS), protein degradation, protein homeostasis, proteotoxic stress, proteasome inhibition, unfolded protein response (UPR)

## Abstract

Numerous cellular processes are controlled by the proteasome, a multicatalytic protease in the cytosol and nucleus of all eukaryotic cells, through regulated protein degradation. The immunoproteasome is a special type of proteasome which is inducible under inflammatory conditions and constitutively expressed in hematopoietic cells. MECL-1 (β2i), LMP2 (β1i), and LMP7 (β5i) are the proteolytically active subunits of the immunoproteasome (IP), which is known to shape the antigenic repertoire presented on major histocompatibility complex (MHC) class I molecules. Furthermore, the immunoproteasome is involved in T cell expansion and inflammatory diseases. In recent years, targeting the immunoproteasome in cancer, autoimmune diseases, and transplantation proved to be therapeutically effective in preclinical animal models. However, the prime function of standard proteasomes and immunoproteasomes is the control of protein homeostasis in cells. To maintain protein homeostasis in cells, proteasomes remove proteins which are not properly folded, which are damaged by stress conditions such as reactive oxygen species formation, or which have to be degraded on the basis of regular protein turnover. In this review we summarize the latest insights on how the immunoproteasome influences protein homeostasis.

## 1. Introduction

The ubiquitin–proteasome-system (UPS) plays an important role in intracellular protein degradation and turnover in the cytosol and nucleus of eukaryotes via a multi-enzymatic machinery, entailing target protein ubiquitination and subsequent proteolysis by the 26S proteasome. The proteasome exerts numerous essential regulatory functions in nearly all cell biological pathways. A protein is usually targeted for degradation by the UPS by the covalent conjugation of poly-ubiquitin chains to one or more of its lysines. Poly-ubiquitin chains containing a minimum of four ubiquitin molecules are efficiently recognized and degraded by the 26S proteasome [1]. The 26S proteasome consists of a 19S regulator bearing ubiquitin receptors, deubiquitylating enzymes, and an ATPase ring in charge of protein unfolding as well as a 20S proteolytic core complex. Structurally, the 20S proteasome core particle consists of α and β subunits that build a barrel-shaped complex of four rings with seven subunits each [2]. The outer two rings consist of α subunits, and the inner two rings of β subunits bearing the catalytically active proteasome subunits PSMB6 (β1c), PSMB7 (β2c), and PSMB5 (β5c). These three proteolytically active subunits are responsible for at least three peptidase activities: caspase-like (exerted by β1c), trypsin-like (exerted by β2c), and chymotrypsin-like (exerted by β5c) activity. In hematopoietic cells and in cells stimulated with IFN-γ, the catalytically active standard proteasome subunits β1c, β2c, and β5c in the inner two rings of the 20S proteasome are replaced by β1i (low molecular mass polypeptide (LMP)2; PSMB9), β2i (multicatalytic endopeptidase complex–like (MECL)-1; PSMB10), and β5i (LMP7; PSMB8), forming the so-called immunoproteasome. Furthermore, mixed structures consisting of standard and immunoproteasome subunits, named intermediate proteasomes, have been described [3]. The immunoproteasome subunit exchanges alter the cleavage-specificity of the 20S proteasome. In immunoproteasomes, the caspase-like activity, exerted by β1c in standard proteasomes, is strongly reduced, whereas the chymotrypsin-like activity is enhanced. This leads to the generation of peptides with hydrophobic C-terminal residues, which are suitable for MHC-I presentation [4]. The involvement of the immunoproteasome in antigen processing influences the pathogen-induced cytotoxic T lymphocyte (CTL) responses, pathogen clearance, and shapes the CTL repertoire [5,6,7,8,9,10]. Apart from MHC-I antigen processing, immunoproteasomes are involved in T cell expansion [7,11,12], T helper cell differentiation [13,14], macrophage polarisation [15,16], in protection from immunopathological damage in the brain [17,18], lung-associated diseases [19,20,21,22], neurodegenerative diseases [23,24,25,26,27,28,29,30,31], and inflammatory diseases [14,32,33,34,35,36,37,38,39] (summarized in [40]). A specific role of the immunoproteasome in NF-κB activation has remained controversial [41,42,43,44,45]. However, using different cell types derived from immunoproteasome-deficient mice it was shown that the immunoproteasome has no specialized function for canonical NF-κB activation [46]. Immunoproteasomes are constitutively expressed in hematopoietic cells, induced in cells stimulated with IFN-γ, and up-regulated in certain cancers. Additionally, due to the role of proteasomes in the regulation of numerous cellular processes and the requirement of immunoproteasome subunits for the survival of T cells in a proinflammatory environment [11], inhibition of the proteasome is an attractive strategy for ameliorating inflammatory diseases. Indeed, different immunoproteasome inhibitors were shown to be effective in pre-clinical models for inflammatory diseases [47,48,49] (summarized in [50,51,52,53]).

## 2. The Proteasome, a Key Player in Protein Homeostasis

Homeostasis is a key determinant of cellular lifespan. Maintenance of cellular protein homeostasis is achieved by balancing protein stability and resistance to stress, protein repair, protein refolding, and proteolysis of damaged proteins. The ratio of protein synthesis and protein breakdown is tightly regulated and adjusted according to intracellular and extracellular cues such as a shortage of amino acid supply for de novo synthesis or activation and growth signalling mediated via surface receptor stimulation. Ribosomes, together with the endoplasmic reticulum (ER), and in close collaboration with multiple co-factors, are the key drivers of protein synthesis, folding, maturation, and sorting, while the proteasome plays a pivotal role in terminating the existence of thousands of proteins that are misfolded, damaged, or otherwise obsolete. A prominent role in proteostasis is ensured by ATP-dependent cellular machineries dedicated to proper protein folding, called chaperones [54]. In eukaryotic cells, two major protein clearance pathways, proteasome and autophagy, exist to maintain cellular proteolysis. The proteasome, in collaboration with the ubiquitylation system, is responsible for clearance of short-lived proteins as well as misfolded or damaged proteins in the cytoplasm, nucleus, and endoplasmic reticulum. By contrast, autophagy, an evolutionarily conserved catabolic pathway, mediates degradation of a large variety of cytosolic substrates, ranging from single proteins to entire organelles or multi-subunit macromolecular complexes.

Dysregulation of normal protein homeostasis can lead to an accumulation of poly-ubiquitylated proteins and oxidized or otherwise damaged proteins in the cell. This can lead to many human illnesses, including neurodegenerative disorders [55], and cancers. The UPS is involved in a range of essential cellular processes, including cell-cycle progression, apoptosis, DNA repair, antigen presentation, inflammation, signal transduction, and protein quality control [56]. The UPS is central to the unfolded protein response (UPR), which is activated when unfolded or misassembled proteins accumulate in the endoplasmic reticulum (ER) [57]. UPR induction results in both an initial decrease in general protein synthesis, to reduce the influx of nascent proteins into the ER, and increased transcription of ER resident chaperones, folding enzymes, and components of the protein degradative machinery to prevent the aggregation of the accumulating misfolded proteins. Misfolded proteins are recognized by ER quality control systems, and if these proteins cannot be properly refolded, they are targeted for ER-associated protein degradation (ERAD) [58]. This involves the retrograde translocation of the misfolded proteins out of the ER and subsequent degradation by cytosolic 26S proteasomes. The classical UPR as an ER-stress response is mediated by three major pathways initiated by three ER transmembrane proteins: inositol-requiring enzyme 1-alpha (IRE1α), protein kinase R (PKR)-like endoplasmic reticulum kinase (PERK), and activating transcription factor 6 (ATF6). IRE1α acts as an mRNA splicing enzyme that selectively splices the mRNA of X-box binding protein (XBP)1, resulting in an active protein product XBP1s from the spliced mRNA. XBP1s induces genes involved in lipid synthesis, ERAD, and protein folding. ATF6 is transported from the ER to the Golgi, where the cytoplasmic domain is cleaved off and translocated to the nucleus to stimulate stress response genes. Activation of PERK by oligomerization and auto-transphosphorylation leads to the phosphorylation of the eukaryotic initiation factor 2α (eIF2α), resulting in decelerated protein synthesis. Furthermore, phosphorylated eIF2α induces activating transcription factor (ATF)4, which activates downstream transcription of metabolic pathways, antioxidant pathways, autophagy, and apoptosis. Interestingly, PERK-dependent phosphorylation directly triggers dissociation of nuclear factor erythroid-derived 2 related factor (Nrf2)/Kelch-like ECH-associated protein 1 (Keap1) complexes and subsequent Nrf2 nuclear import [59]. In the nucleus, it binds to antioxidant response elements (ARE) in the promotor regions of genes of the UPS. Among others, mature Nrf2 activates transcription of all genes encoding subunits of the 26S proteasome. This results in the assembly of more 26S proteasomes, which allows cells to escape the cytotoxic effects of accumulating proteins.

The important role of the proteasome in protein homeostasis is strikingly shown in cells treated with proteasome inhibitors. The primary, immediate consequence of proteasome inhibition is a decrease in overall rates of protein breakdown in cells leading to a rapid accumulation of short-lived proteins conjugated to ubiquitin and misfolded/damaged proteins which may constitute a large fraction (up to one-third) of newly synthesized polypeptides (also designated ‘defective ribosomal products’ (DRIPs) [60]. The accumulation of these polypeptides triggers the expression of heat shock proteins and the activation of the UPR. Depending on the cell type and proliferation state, the accumulation of poly-ubiquitylated proteins might trigger apoptosis in proteasome inhibited cells.

## 3. Immunoproteasomes in Protein Homeostasis. Contradictory Results

The indispensable role of the proteasome in maintaining protein homeostasis is undisputed. However, is there a specialised function of immunoproteasomes in preventing accumulation of ubiquitylated proteins, making the immunoproteasome superior to standard proteasomes?

The requirement for immunoproteasomes in the degradation of proteins in steady state in vivo in mice seems not to be essential. Naïve mice devoid of all three immunoproteasome subunits did not show accumulation of ubiquitylated proteins in the spleen, an organ expressing high levels of immunoproteasomes in wild-type mice [61]. Hence, under non-inflammatory conditions, standard proteasomes seem to handle protein degradation without support of immunoproteasomes. This could be confirmed in splenocytes derived from LMP7-deficient mice in a recent study from our laboratory [62]. Additionally, activated B cells derived from LMP2-deficient mice vs. wild-type mice have no significant difference in the levels of poly-ubiquitylated proteins [63]. Furthermore, de Verteuil et al. neither found a difference in poly-ubiquitylated proteins in immature LMP7/MECL-1 double deficient dendritic cells (DCs) nor in LPS-stimulated DCs compared with wild-type DCs [64]. Similarly, Hewing et al. did not observe differences in poly-ubiquitin conjugate clearance in bone marrow derived macrophages from WT or LMP7-deficient mice either in unstimulated macrophages or after IFN-γ exposure [65].

Cells stimulated with IFN-γ, a cytokine mainly produced under inflammatory conditions by T cells and natural killer cells, revealed a significant enrichment of poly-ubiquitin conjugates between 4 and 12 h after IFN-γ stimulation [66]. This enrichment appeared to be a transient response because the levels of detectable poly-ubiquitin conjugates initially increased and later returned to reduced levels between 24 and 48 h of IFN-γ stimulation. The accumulation of poly-ubiquitin conjugates was detectable in both mouse and human cells independent of the cell type [66]. Cycloheximide chase experiments demonstrated that the increase in poly-ubiquitin protein levels was strongly dependent on translation, suggesting that primarily newly translated proteins shape the pool of poly-ubiquitin substrates in response to IFN-γ. Furthermore, the same experiments also revealed that poly-ubiquitylated proteins are degraded significantly faster in IFN-γ-treated than in untreated cells [66]. During the early IFN-γ response the E2 conjugation enzyme UBE2L6-dependent ubiquitin conjugation activity plays an important role in accumulation of ubiquitin conjugates. In addition to enhanced ubiquitylation activity, IFN-γ stimulation resulted in a transient decrease in proteasomal chymotrypsin-like activity 4–12 h after induction, which was restored and even enhanced after 24–48 h [66]. Among other proteins, the immunoproteasome is strongly induced in IFN-γ treated cells. The time frames exhibiting the highest accumulation of poly-ubiquitin conjugates in these experiments corresponded exactly with the decline of proteasomal peptidase activity and coincided with the time period required for the formation of immunoproteasomes [66]. This would indicate that immunoproteasomes are more efficient in degrading poly-ubiquitylated proteins than standard proteasomes. Indeed, whereas in wild-type cells a transient accumulation of poly-ubiquitin conjugates that returned to basal levels at 48 h after IFN-γ challenge was observed, in LMP7-deficient murine embryonic fibroblasts (MEF) and in a human cell devoid of LMP2/7, the accumulation of poly-ubiquitin conjugates persisted over the entire period without returning to basal levels [66]. Misfolded proteins can be directed into cytoplasmic aggregates such as aggresomes and aggresome-like induced structures (ALIS) to avoid potentially toxic effects of accumulated poly-ubiquitin conjugates [67,68,69]. In line with a crucial role of immunoproteasomes in degrading poly-ubiquitylated proteins, LMP7-deficient MEFs showed a high accumulation of ALISs in immunoproteasome-deficient cells at 48 h post IFN-γ treatment, a time point at which wild-type cells were already cleared from ALISs [66]. Furthermore, in the liver of LPS-treated mice or in the brain of experimental autoimmune encephalomyelitis (EAE)-diseased mice, an increased accumulation of poly-ubiquitin conjugates could be observed in LMP7-deficient mice compared with the wild-type mice [66]. Taken together, the results of Seifert et al. showed that cells devoid of immunoproteasomes are unable to efficiently degrade accumulating poly-ubiquitin conjugates in response to IFN-γ, both in cell culture studies and in in vivo inflammation models [66].

However, contradictory results were obtained in the study by Nathan et al. [70]. No transient increase in ubiquitin conjugates were formed in murine and human cells between 4 and 12 h after IFN-γ treatment, although the immunoproteasome was induced. Proteasome activity studies using small fluorogenic peptides for chymotrypsin- and caspase-like activity did not reveal a transient decrease in proteasome activity in response to IFN-γ. Furthermore, no decrease in total proteasome content could be observed after IFN-γ treatment. Purified standard 26S proteasome and purified 26S immunoproteasome were used to investigate whether immunoproteasomes are superior in degrading poly-ubiquitylated proteins. Degradation studies using ubiquitylated dihydrofolate reductase (Ub_5_DHFR) showed that 26S immunoproteasomes and 26S standard proteasomes degrade poly-ubiquitylated conjugates at similar rates. Furthermore, no difference in the abilities of the standard and the immuno-26S-proteasomes to bind to poly-ubiquitin chains was observed. This is consistent with prior findings that the 20S and 26S immunoproteasomes degrade denatured ovalbumin at the same rates as the corresponding standard particle [71]. Poly-ubiquitylated E6AP similarly activated peptide hydrolysis of 26S standard proteasomes and 26S immunoproteasomes [70]. In further experiments, the accumulation of poly-ubiquitylated proteins was investigated in the absence of the immunoproteasome subunit LMP7. Poly-ubiquitin conjugate formation in MEFs derived from wild-type mice and LMP7^−/−^ mice was similar, and no transient poly-ubiquitin conjugate accumulation was observed in response to IFN-γ. Formation of ALIS in response to IFN-γ was similar in wild-type MEFs and LMP7-deficient MEFs. Taken together, the findings by Nathan et al. do not support the conclusion that 26S immunoproteasomes are more efficient than standard particles in degrading poly-ubiquitylated proteins [70].

In agreement with the study by Nathan et al. [70], Abi Habib and colleagues found that the efficiency to degrade ubiquitylated proteins is similar between standard proteasomes, immunoproteasomes, and intermediate proteasomes [72]. In cells that exclusively express one type of proteasome, degradation rate of ubiquitylated p21 and ubiquitylated c-myc was similar, indicating that different types of 26S proteasomes degrade ubiquitylated proteins equally well.

After infection of mice with virus [62,73], fungi [74], or bacteria [75], standard proteasomes are rapidly replaced in infected organs by newly formed immunoproteasomes. The up-regulation of immunoproteasomes has mainly been associated with improved generation of ligands for MHC-I antigen presentation. However, the main function of proteasomes within cells is to maintain protein homeostasis by selectively degrading proteins. Hence, the up-regulation of immunoproteasomes during infection might have been evolved to accomplish the higher proteolyical demand under inflammatory conditions. In the course of a viral infection, virus-infected cells are hijacked by the virus to massively produce virus progenitors. Furthermore, immune cells are activated to combat the foreign intruder. Replication of viruses and activation of immune cells leads to enhanced production of newly synthesized proteins and thus to increased protein degradation to maintain protein homeostasis. Indeed, activation of T cells leads to a strong increase in the rate of protein synthesis [76,77]. Using virus-specific T cells it was shown that viral infection in mice led to an accumulation of poly-ubiquitylated proteins in CD8^+^ T cells, 24 h post infection [62]. This indicates that the increase in translation rate in activated T cells results, at least transiently, in an enhanced proteolytic demand. However, the total proteasome amount in the liver and spleen is not increased. In that study, it was further investigated whether the immunoproteasome is critically required to cope with the altered challenges in protein degradation during a viral infection [62]. For this, the well-studied lymphocytic choriomeningitis virus (LCMV) was used. The non-cytopathic arenavirus LCMV induces a strong cytotoxic T cell response, which is essential for the elimination of the virus from infected mice. LCMV infection induces a strong up-regulation of immunoproteasomes in the liver on day 8 post infection, an organ that, in naïve state, barely expresses immunoproteasomes [62,73]. In contrast, in the spleen, an organ which contains mainly immunoproteasomes in naïve mice, the immunoproteasome is not induced [62]. LMP7-deficient mice hardly show any incorporation of MECL-1 and LMP2 into proteasomes in the liver and spleen after LCMV infection. Hence, the almost complete lack of immunoproteasome induction in LMP7-deficient mice renders these mice an ideal model in which to investigate the requirement of immunoproteasomes to cope with the enhanced degradation of proteins during viral infection. Analysis of poly-ubiquitin in the spleen and liver of LCMV-infected wild-type mice and LMP7-deficient mice on days 3, 5, and 7 post LCMV infection revealed no difference in poly-ubiquitin accumulation between these mice. Furthermore, accumulation of proteins in activated T cells, if not resolved, would finally lead to the induction of apoptosis. However, apoptosis induction, as measured by PARP cleavage, was not enhanced in the liver and spleen of LCMV-infected LMP7-deficient mice. These data indicate that in the course of a viral infection, immunoproteasome activity is not required to cope with the increased need for protein degradation during virus replication and T cell activation [62].

The role of the immunoproteasome during viral infection was also investigated in LMP7-deficient mice in acute coxsackievirus B3 (CVB3) myocarditis [78]. The immunoproteasome is rapidly induced in cardiac tissue post CVB3 infection. Although similar viral titres were observed in cardiac tissues of wild-type mice and LMP7^−/−^ mice, increased immune cell infiltration into these tissues and an exacerbated acute heart muscle injury was observed in LMP7^−/−^ mice. To investigate the role of immunoproteasomes on cellular protein equilibrium in this study, primary cardiomyocytes and B cell depleted splenocytes from wild-type mice or LMP7^−/−^ mice were exposed to IFN-γ in vitro. The lack of LMP7 coincided with increased accumulation of poly-ubiquitylated proteins post IFN-γ stimulation. Consistent with this result, LMP7-deficient mice failed to cope with accelerated protein turnover in CVB3 infected mice, as reflected by increased accumulation of poly-ubiquitylated proteins in cardiac tissues of diseased mice. Furthermore, oxidant protein damage was increased in acutely inflamed hearts in LMP7-deficient mice. Taken together, these data support the role of immunoproteasome formation in cardiomyocytes and in inflammatory cells in protecting the diseased tissue from proteotoxic stress during acute CVB3 infection [78].

Apart from a special role of immunoproteasomes in degradation during inflammation, their role was also investigated in steady state level in the thymus [79]. Medullary thymic epithelial cells (mTECs) contribute to self-tolerance through the promiscuous expression of tissue-specific antigens in the thymus, which mediate negative selection of self-reactive T cells [80]. mTECs can be subdivided into different subsets according to their MHC-II expression. Most promiscuous gene expression occurs in mTECs showing high MHC-II expression (mTEC^high^). Compared with other cell types in the thymus, mTEC^high^ cells synthesize significantly more proteins [79]. Cells with increased protein synthesis rate are prone to higher sensitivity to proteotoxic stress. To cope with proteotoxic stress, cells activate the UPR. Although some factors of the UPR are activated in mTEC^high^ cells, these cells are not in an apoptotic state [79]. Increasing the chaperone activity or the protein degradation are two strategies for preventing proteotoxic stress. St-Pierre et al. hypothesized that constitutive expression of immunoproteasomes in mTECs could be important for maintaining protein homeostasis [79]. On an mRNA level, immunoproteasome subunits are expressed in mTECs [79,81]. The role of immunoproteasome subunits in maintaining protein homeostasis in mTECs was investigated in LMP7/MECL-1-double deficient (dKO) mice [79]. Compared with wild-type mice, the size of medullary regions in thymi of dKO mice was reduced, accompanied by a marked decrease in mTEC abundance [79]. Using bone marrow chimeras, it was shown that the loss of mTECs in dKO mice was an mTEC cell-intrinsic phenomenon. mTEC^high^ cells in dKO mice have a reduced half-life, accompanied by an increased proportion of apoptotic cells. Although wild-type and dKO TECs contain similar numbers of TEC progenitors, these progenitors have lost their ability to generate mTECs in dKO mice. Analysis of proteotoxic stress in mTECs revealed selective activation of PERK signalling in mTEC^high^ of adult dKO mice, manifested by increased levels of phospho-eIF2α leading to a decrease in protein synthesis rate. Treatment of foetal thymic organ cultures (FTOC) with tunicamycin to induce proteotoxic stress led to an approximately 2-fold higher reduction in the number of mTECs in FTOC cultures derived from dKO mice compared with wild-type mice. Compared with tunicamycin-treated wild-type mTECs derived from FTOC cultures, central mediators of the UPR and their induced genes were more drastically up-regulated in dKO mTECs. However, the heat shock response, as determined by the analysis of heat-shock factor 1 (HSF1), was not altered in dKO mTECs. In line with a decreased cellularity of mTECs of tunicamycin-treated FTOCs derived from dKO mice, the pro-apoptotic genes Bad and Bax were up-regulated in these cells. Taken together, the authors conclude from these results that immunoproteasomes play a non-redundant role in mTECs by alleviating proteotoxic stress [79].

Acute pancreatitis is a primarily sterile disease thought to be induced due to premature intra-acinar activation of digestive pancreatic zymogens [82]. Among others, impairment of the UPR was found to be one factor that contributes to the disease [83]. The caerulein in vivo mouse model of pancreatitis induces a mild and reversible form of the disease. Caerulein injection rapidly induces pancreatic enzyme activation leading to disease symptoms similar to those of acute pancreatitis in humans, such as hyperamylasaemia, infiltration of inflammatory cells within the pancreas, pancreatic oedema, acinar cell vacuolization, and the presence of activated pancreatic enzymes within the pancreas [84]. In this model, a higher pancreatic damage can be observed in LMP7-deficient mice [38]. Furthermore, compared with wild-type mice, LMP7-deficient mice show increased activity of pancreatic enzymes in the acute phase of the disease as well as enhanced activation of pro-inflammatory mediators. At 8 h post induction, acute pancreatitis is associated with an accumulation of poly-ubiquitylated proteins in the pancreas, which is enhanced in LMP7-deficient mice. These ubiquitin-conjugates, which were resolved 24 h post induction, were exclusively detected in acini but not in macrophages or neutrophils. These data might indicate that the absence of LMP7 has an impact on protein degradation during acute pancreatitis. Hence, impaired protein degradation might result in increased ER stress and activation of the UPR. Indeed, CHOP protein levels were significantly enhanced at 24 h in the pancreas of LMP7-deficient mice. Furthermore, cholecystokinin treated in vitro cultures of pancreatic acini lacking LMP7 showed increased levels of different ER stress response transcripts. Taken together, these findings indicate that the immunoproteasome plays a protective role in acute pancreatitis by clearing damaged proteins and balancing ER stress responses [38].

## 4. The Role of the Immunoproteasome in Degrading Oxidized Proteins

Formation of reactive oxygen species (ROS) can be triggered by environmental factors, such as certain drugs, toxins, or ionizing radiation. Furthermore, biochemical processes within cells lead to the formation of ROS. Normal mitochondrial ATP production by oxidative phosphorylation produces superoxide anions as a by-product, which can be converted to hydrogen peroxide [85]. To kill intracellular bacteria, macrophages produce ROS by NADPH-oxidase, a process called respiratory burst [86]. Family members of the NADPH-oxidase complex (Nox) are inducible in a wide range of cell types. Under inflammatory conditions, oxidative stress is promoted by IFN-γ by inducing Nox isoforms, such as Nox1, resulting in increased ROS production [87]. To counteract damage to cells by ROS, canonical antioxidant mechanisms in cells are induced. This includes induction of ROS-alleviating enzymes such as superoxide dismutase or ROS scavenging molecules such as glutathione. If the cell cannot counteract the oxidizing effects of ROS, the resulting imbalance is called ‘oxidative stress’. Under oxidative stress, 26S proteasomes seem to be unstable and dissociate in 20S proteasomes and 19S regulators. Hence, degradation of oxidized proteins possibly occurs independent of the 19S regulatory particle and the ubiquitin system [72,88,89,90]. The loss of protein structure rather than the presence of oxidized residues targets oxidized proteins to the catalytic chamber of 20S proteasomes [72,91]. Immunoproteasomes were reported to be up-regulated by oxidative stress after exposure to H_2_O_2_ [92] or nitric oxide species [93]. The nitric oxide induced up-regulation of immunoproteasomes enhanced proteasome activity which was abrogated in cells transfected with antisense LMP2 and LMP7 oligonucleotides, indicating that immunoproteasomes might protect cells from oxidative stress [93]. In the presence of oxidative stress and upon proteasome dysfunction the activation of the Nrf2 pathway is induced. Under oxidative stress conditions the destabilizing conformational influence of Keap1 on Nrf2 is disrupted. Subsequently, Nrf2 can accumulate and translocate to the nucleus, where it binds to antioxidant response elements (ARE) in the promotor regions of many genes, including standard proteasome subunits [92,94]. Immunoproteasome subunits were not found to be regulated by Nrf2 even though *Psmb8*/LMP7 contains the Nrf2-responsive ARE consensus sequence upstream of the gene [92,94]. Hence, how immunoproteasome expression is regulated under oxidative stress remains elusive.

Upon IFN-γ stimulation, ROS levels increase in HeLa and T1 cells, leading to increased levels of oxidized proteins [66]. In agreement, the antioxidant sulforaphane impaired the IFN-γ-induced accumulation of oxidant-damaged proteins. In this study, the amount of oxidized proteins coincided with increased amounts of poly-ubiquitin conjugates and was significantly higher in cells lacking immunoproteasomes, indicating that immunoproteasomes play a crucial role in removing oxidized proteins. To directly investigate the impact of immunoproteasomes on degradation of oxidant-damaged proteins, wild-type and LMP7-deficient skin fibroblasts were exposed to IFN-γ. Levels of oxidized proteins in wild-type cells were transiently increased between 8 and 24 h of IFN-γ exposure, whereas LMP7-deficient fibroblasts exhibited significant amounts of oxidized proteins throughout the time course, with the highest accumulation at 48 h post IFN-γ exposure. Notably, such a transient induction of oxidant-damaged proteins was also detectable in vivo in liver tissue 24 h post LPS-induced inflammation. In contrast with wild-type mice, LMP7-deficient mice failed to clear the accumulation of oxidized proteins in the liver 48 h after LPS challenge. These data indicate that proteins that are oxidant damaged by IFN-induced ROS become a substrate of the immunoproteasome. IFN-γ-induced oxidative stress may eventually result in apoptosis-induced cell death. Indeed, compared with wild-type cells, LMP7-deficient cells are prone to apoptosis with elevated caspase 3/7 activity in response to IFN-γ and are more susceptible to the apoptosis inducer etoposide [66].

Primary cardiomyocytes and B cell depleted splenocytes deficient for LMP7 show an increased accumulation of oxidant-damaged proteins in response to IFN-γ [78]. Oxidant protein damage was increased in acutely inflamed hearts in CVB3-infected LMP7-deficient mice compared with wild-type mice [78].

In contrast with these studies [66,78], Hewing et al. found no difference in poly-ubiquitin conjugate clearance in bone marrow-derived macrophages from wild-type mice and LMP7-deficient mice after exposure to H_2_O_2_, a potent inducer of oxidative stress [65].

Abi Habib et al. evaluated the ability of different proteasome subtypes (standard proteasome, immunoproteasome, and intermediate proteasomes) to degrade oxidized proteins [72]. The different types of 20S proteasomes were not able to degrade native calmodulin since folded proteins are generally targeted to 26S proteasomes in a ubiquitin-dependent manner. However, H_2_O_2_-oxidized calmodulin was degraded by all types of 20S proteasomes, with intermediate proteasomes and immunoproteasomes degrading oxidized calmodulin faster than the standard proteasome. Similar results were obtained with oxidized haemoglobin, indicating that LMP7-containing 20S proteasomes more rapidly degraded oxidized proteins. However, the presence of oxidized methionines does not explain the different degradation rate of oxidized proteins by the different proteasome subtypes. Interestingly, similar to oxidized calmodulin or hemoglobulin, the intrinsically disordered protein tau was degraded faster by LMP7-containing 20S proteasomes in a ubiquitin-independent manner. Taken together, the study by Abi Habib et al. suggest that LMP7-containing proteasomes play a key role in the clearance of disordered and oxidatively damaged proteins [72].

## 5. Immunoproteasome Inhibition and Protein Homeostasis

**Figure 1:**
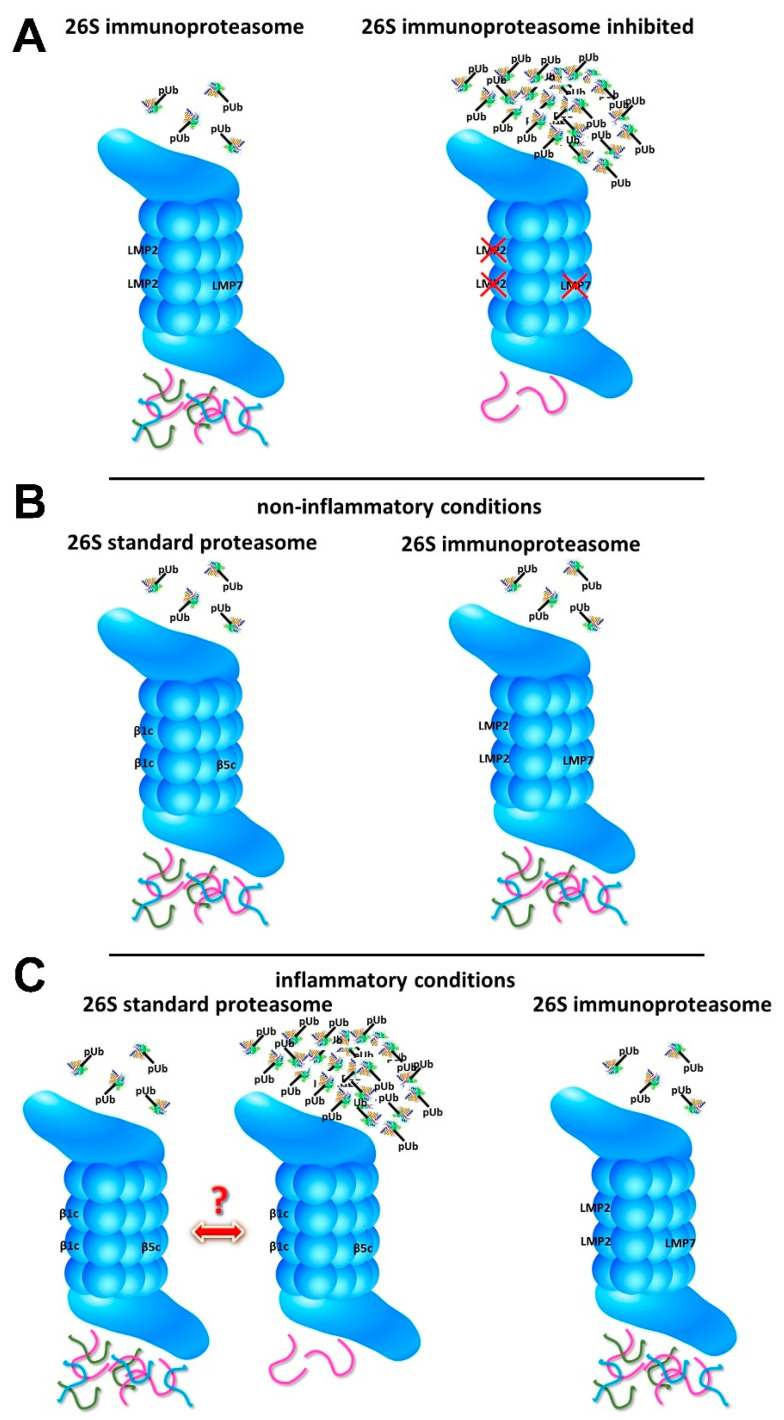
Is the immunoproteasome required to degrade poly-ubiquitylated proteins under inflammatory conditions? (**A**) 26S immunoproteasomes degrade poly-ubiquitylated (pUb) proteins into peptides (**left side**). In activated immune cells or in plasma cells, inhibition of at least two proteolytically active immunoproteasome subunits leads to an accumulation of poly-ubiquitylated proteins (pUb) (**right side**). (**B**) Under non-inflammatory conditions 26S standard proteasomes (**left side**) and 26S immunoproteasomes (**right side**) can degrade poly-ubiquitylated proteins (pUb) into peptides and maintain protein homeostasis. (**C**) 26S immunoproteasomes degrade poly-ubiquitylated proteins (pUb) into peptides (**right side**). Whether 26S standard proteasomes can maintain protein homeostasis also under inflammatory conditions remains controversial (**left side**). If 26S standard proteasomes cannot cope with the increased proteolytical demand under inflammation, it leads to an accumulation of poly-ubiquitylated proteins (pUb). The third immunoproteasome subunit MECL-1 is located on the back of the 26S proteasome and is therefore not depicted in the 26S proteasome schemes.

In contrast with cells lacking immunoproteasomes, the effect of immunoproteasome inhibition on protein homeostasis is mechanistically rather different. While the lack of immunoproteasome subunits, might decelerate the degradation of proteins, blockage of immunoproteasome subunits leads to a reduction in degradation capacity that depends on the degree of inhibition. One or multiple active sites of the immunoproteasome are blocked by inhibitors, leading to a reduced degradation of proteins (Figure 1A). Proteasome inhibitors are clinically approved for the treatment of myeloma and mantle cell lymphoma. Proteasome inhibition in multiple myeloma causes a toxic build-up of proteins leading to apoptosis [95]. The more proteasome subunits are targeted, the less proteins can be degraded by the proteasome, leading to an accumulation of proteins destined to be degraded. The cell counteracts by the induction of the UPR and by the expression of newly synthesized 26S proteasome components. If the cell does not succeed, apoptosis is induced in proteasome-inhibited cells. It has been shown that both β5 and β2 have to be targeted to induce cell death in solid tumours [96] (summarized in [51]). β5 and β2 co-inhibition causes aggregation of Nrf1, which blocks up-regulation of proteasome genes and prevents recovery of proteasome activity, and, thus, leads to cell death in solid tumours. An inhibitor which shows such a β5 and β2 profile is the recently described glidobactin C [97]. Inhibition of a single immunoproteasome subunit seems not to be sufficient to have a positive effect in autoimmunity. LMP7 and LMP2 or LMP7 and MECL-1 have to be co-inhibited to ameliorate rheumatoid arthritis, inflammatory colitis, and experimental autoimmune encephalomyelitis in animal models [98,99] (summarized in [51]).

ONX 0914 (formerly designated PR-957) is an irreversible proteasome inhibitor that selectively targets the immunoproteasome subunits LMP7 and LMP2 [47,98]. In contrast with other human immune cells, such as B cells, T cells, or natural killer cells, ONX 0914 selectively kills human blood-derived CD14^+^ monocytes [100]. The high immunoproteasome expression in CD14^+^ monocytes predisposes these cells to ONX 0914 treatment because both LMP2 and LMP7 are blocked by ONX 0914. This leads to a perturbation in protein turnover, indicated by an accumulation of poly-ubiquitylated proteins and the induction of the UPR. The monocytes finally die due to the induction of apoptosis. Interestingly, shutting off protein synthesis with the translation inhibitor cycloheximide protected CD14^+^ monocytes from ONX 0914-induced cell death, indicating that newly synthesized proteins contribute to the accumulation of proteins in immunoproteasome-targeted monocytes. Compared with CD4^+^ T cells, CD14^+^ cells are translationally more active, probably rendering these cells especially sensitive to immunoproteasome inhibition. Since monocytes are the main producers of IL-23 and immunoproteasome inhibition selectively induces apoptosis in monocytes [100], immunoproteasome inhibition might be a strategy for targeting the central IL-23/IL-17 immune axis in autoimmunity.

Because of their extremely high rate of antibody synthesis, plasma cells are particularly sensitive to proteasome inhibition. Proteasome inhibition in these cells causes an accumulation of misfolded proteins within the endoplasmic reticulum, thereby activating the terminal UPR, leading to apoptosis. Targeting plasma cells in animal models for systemic lupus erythematosus and kidney transplantation has been successful with the immunoproteasome inhibitor ONX 0914 [101,102]. Plasma cells from kidney allotransplanted rats express immunoproteasomes at high levels [103]. For that reason, immunoproteasome inhibition resulted in a significant accumulation of poly-ubiquitylated protein conjugates in plasma cells in vivo, followed by the activation of the UPR and the induction of apoptosis in plasma cells [103]. Hence, selective immunoproteasome inhibition is a promising pharmacologic approach for interfering with plasma cell survival that prevents chronic, antibody-mediated renal allograft rejection.

Immunoproteasome inhibition alters T cell activation, T cell differentiation, and cytokine secretion of activated immune cells [104]. Naïve mouse B cells and T cells mainly express immunoproteasomes [105]. Since standard proteasomes are lacking in these cells, immunoproteasome inhibition reduces cleavage capacity of virtually all proteasomes in these cells. Schmidt et al. investigated the effect of immunoproteasome inhibition on protein homeostasis in early lymphocyte activation [105]. While CD4^+^ T cells treated with the pan proteasome inhibitor MG-132 immediately showed ubiquitin conjugate accumulation, ONX 0914-treated cells did not show such effects at early time points. Nevertheless, ONX 0914 treatment induced a robust accumulation of ubiquitin-conjugates after 3–4 h post T cell receptor-triggered activation. Interestingly, LMP7-deficient cells did not show ubiquitin accumulation, showing that standard proteasomes can cope with the increased proteolytic demand during early T cell activation. Notably, when cells were left unstimulated, many fewer ubiquitin-conjugates were detected, indicating that the bulk of proteostatic stress was activation-induced and not due to steady-state proteostasis. Treatment with the protein synthesis inhibitor cycloheximide abolished ubiquitin conjugate accumulation in ONX 0914-treated cells, supporting findings that protein neosynthesis was the primary driver of proteostatic stress after immunoproteasome inhibition. To study the consequence of the observed ubiquitin-conjugate accumulation, cell function and survival were analysed. In contrast with MG-132, ONX 0914 treatment did not induce stress response pathways resulting in p53 accumulation, ATF4 induction, and eIF2a-phosphorylation in activated T cells. Furthermore, analysis of PARP cleavage did not show enhanced apoptosis in activated mouse and human T cells. ONX 0914-treated activated T cells retained viability even 20 h after activation. Interestingly, ubiquitin conjugates in ONX 0914-treated T cells accumulating between 3 and 4 h post activation disappeared within 20 h. This was not attributed to LMP7 up-regulation but to increased β5c protein levels, which correlated with Nrf1 expression, indicating that proteostatic stress in immunoproteasome-inhibited cells might be alleviated via standard proteasome up-regulation. How mild proteostatic stress in ONX 0914-treated T cells might lead to reduced T cell activation, T cell differentiation, and cytokine secretion remains to be investigated.

Winter et al. investigated the effect of immunoproteasome inhibition on the UPS in the B-lymphoblast cell line SUP-B15 [106]. SUP-B15 has an LMP7-to-β5c activity ratio of 80% to 20%. Inhibition of LMP7 with ONX 0914 led to a rapid accumulation of poly-ubiquitylated proteins, while inhibition of β5c with PR-825 had no effect. Accumulation of poly-ubiquitylated proteins in ONX 0914-treated SUP-B15 cells correlated with induction of LMP7 on mRNA and protein level. Furthermore, similar to the observation made by Schmidt et al. [105], a compensatory increase in *PSMB5* mRNA and β5c protein levels was observed in ONX 0914-treated SUP-B15 cells, which went along with an induction of Nrf1 [106]. However, although β5c was up-regulated on protein levels, an increase in β5c activity was not observed.

## 6. Concluding Remarks

A crucial role of the proteasome in maintaining protein homeostasis is undisputed. However, a specialised function of the immunoproteasome in degrading proteins making it superior to standard proteasomes remains unclear and controversial. Under steady-state conditions in a non-inflammatory environment, the immunoproteasome seems not to be necessary for maintaining protein homeostasis (Figure 1B). Different mice devoid of single- or multiple-immunoproteasome subunits are viable and show no obvious impairments. Furthermore, compared with wild-type mice, no accumulation of poly-ubiquitin conjugates could be observed [61,66,70,107]. However, these mice are held under laboratory conditions in a specific pathogen-free environment. Nevertheless, the immunoproteasome has evolved in mammals permanently exposed to various environmental influences, such as radiation, temperature changes, and pathogens. Whether immunoproteasome is crucial in maintaining protein homeostasis in the wild remains to be investigated. The role of the immunoproteasome in maintaining protein homeostasis under inflammatory conditions is controversial [38,62,65,66,70,72,78,79] (Table 1) (Figure 1C). In particular, the discrepancy between the two studies of Seifert et al. and Nathan et al. [66,70], who performed rather similar experiments, remains elusive. However, differences between standard- and immunoproteasome in degrading poly-ubiquitylated proteins are difficult to bring in line with our understanding of proteasome function since the capacity to bind and unfold ubiquitylated proteins are functions of the 19S regulatory particles. As poly-ubiquitylated proteins bind initially to the 19S ubiquitin receptors Rpn1, Rpn10, and Rpn13 subunits, which do not differ between the immuno- and standard 26S, it is unclear how changes in the active site specificities of the core 20S particle would alter its ability to degrade poly-ubiquitylated proteins. Structural analysis of the binding surfaces of the 20S core particle for the 19S regulator both of the immuno- and standard 20S proteasome shows high similarity [2]. Hence, the gating mechanisms for substrate entry are unlikely to differ. A different association of standard- and immunoproteasomes with other regulatory particles might be a reason for altered degradation of poly-ubiquitylated proteins. However, no difference in the binding affinity of the 20S immunoproteasome and standard proteasome to the proteasome activator PA28αβ could be observed [108]. A new study using hydrogen–deuterium exchange coupled to mass spectrometry (HDX-MS) describes the existence of allosteric differences between the standard proteasome 20S and the immunoproteasome 20S at the surface of the α-ring triggered from inside the catalytic β-ring [109]. The central pore of the 20S was more flexible in immunoproteasomes. Furthermore, binding of PA28αβ or PA28γ induces conformational changes in the β-rings, the proteasomal active sites, and therefore might modify the 20S products. In addition, the cryo-EM structure of mammalian PA28αβ–immunoproteasome 20S complex suggests that this complex has experienced profound remodelling during evolution to achieve its current level of function in immune response [110]. These data indicate that the binding of regulators to the 20S proteasome allosterically modifies 20S proteasome activity. Whether the 19S regulator, which binds and unfolds ubiquitylated proteins, differently affects the activity of 20S standard proteasomes compared with 20S immunoproteasomes remains to be investigated. It should be kept in mind, however, that the unfolding of proteasome substrates by the 19S regulator is the rate limiting step for protein degradation by the 26S proteasome and not peptide bond cleavage within the 20S core cylinder [111]. This recent insight casts doubt on whether differences in peptide cleavage specificities and rates between the standard and immuno-20S proteasome can account for differences in protein degradation in cells unless their differential docking to the 19S regulator affects its speed of protein unfolding or conformational status. This conception also renders the interpretation of experiments involving protein degradation by 20S standard vs. immuno-proteasomes in vitro difficult with respect to their physiological significance.

Under inflammatory conditions, IFN-γ can promote oxidative stress or induce the UPR [87,112]. Thus, enhanced inflammation seen in immunoproteasome-deficient mice [38,78] might lead to an increased accumulation of poly-ubiquitylated proteins. Hence, the observed increase in poly-ubiquitin conjugates in diseased mice might not be due to an impaired protein degradation but rather due to IFN-γ-induced effects.

Experiments performed in yeast identified the β5 subunit of the proteasome as the rate-limiting subunit in proteasomal protein degradation [113,114]. However, blocking β5 activity in multiple myeloma cells has little effect on protein degradation, proteotoxic stress induction, and cytotoxicity [115]. More than 50% inhibition of protein degradation is achieved only when both β5 and either β1 or β2 sites are inhibited [116,117]. Thus, how the exchange of β5c to LMP7 can influence protein degradation in such a way that in the absence of LMP7 (note that in this case β5c is incorporated into proteasomes exerting β5 cleavage) the degradation capacity of the 20S proteasome cannot cope with enhanced degradation during inflammation or in mTECs (as seen in [38,66,78,79]), when even the complete blockage of the β5 site has no effect on protein degradation, remains rather difficult to explain. Notably, because multiple myeloma cells are highly active in antibody production, they are heavily dependent on a high proteasome capacity to degrade large amounts of misfolded antibody chains, making it astonishing that blocking the β5 site is not sufficient to induce proteotoxic stress. Hence, mechanisms that show how immunoproteasomes contribute to the avoidance of proteotoxic stress during inflammation need to be discovered.

## Figures and Tables

**Table 1:** Summary of studies investigating the effect of immunoproteasomes on protein homeostasis.

Effect on Protein Homeostasis	Inflammatory Condition	Cell Type, Organ, Proteasome Type	Effect on Protein Degradation	Reference
no	naïve mice	spleen of triple KO mice vs. wild-type mice	no accumulation of ubiquitylated proteins	[61]
no	unstimulated or LPS stimulated	LMP^−/−^ vs. wild-type B cells	no accumulation of ubiquitylated proteins	[63]
no	immature DCs, LPS-matured DCs	LMP7^−/−^MECL-1^−/−^ vs. wild-type DCs	no accumulation of ubiquitylated proteins	[64]
no	unstimulated or IFN-γ stimulated	LMP7^−/−^ vs. wild-type macrophages	no accumulation of ubiquitylated proteins	[65]
yes	IFN-γ stimulated	LMP7^−/−^ vs. wild-type MEFs	accumulation of poly-ubiquitylated proteins	[66]
			ALIS formation	
	LPS injection	liver of LPS stimulated LMP7^−/−^ mice vs. wild-type mice	accumulation of poly-ubiquitylated proteins	
	experimental autoimmune encephalomyelitis	brain of diseased LMP7^−/−^ mice vs. wild-type mice	accumulation of poly-ubiquitylated proteins	
no	IFN-γ stimulated	LMP7^−/−^ vs. wild-type MEFs	no accumulation of poly-ubiquitylated proteins	[70]
			no ALIS formation	
		purified 26S standard proteasomes or 26S immunoproteasomes	no difference of 26S standard or 26S immunoproteasomes in degrading Ub_5_DHFR	
no		purified 26S proteasome of cells expressing standard proteasomes, intermediate proteasomes, or immunoproteasomes	efficiency to degrade ubiquitylated proteins is similar between different types of proteasomes	[72]
no	naïve mice	spleen or liver of LMP7^−/−^ mice vs. wild-type mice	no accumulation of poly-ubiquitylated proteins	[62]
	LCMV-infected mice	spleen or liver of diseased LMP7^−/−^ mice vs. wild-type mice	no accumulation of poly-ubiquitylated proteins on d3, 5, 7 post infection	
yes	IFN-γ stimulated	primary cardiomyocytes or B cell depleted splenocytes of LMP7^−/−^ mice vs. wild-type mice	accumulation of poly-ubiquitylated proteins	[78]
	CVB3-infected mice	cardiac tissue of diseased LMP7^−/−^ mice vs. wild-type mice	accumulation of poly-ubiquitylated proteins in cardiac tissues of diseased mice	
yes	unstimulated	mTECs of LMP7^−/−^/MECL-1^−/−^ mice vs. wild-type mice	induction of UPR	[79]
yes	acute pancreatitis	pancreas of diseased LMP7^−/−^ mice vs. wild-type mice	accumulation of poly-ubiquitylated proteins in pancreas	[38]

## Data Availability

Not applicable.
